# Early C-reactive protein kinetics predicts immunotherapy response in non-small cell lung cancer in the phase III OAK trial

**DOI:** 10.1093/jncics/pkad027

**Published:** 2023-04-02

**Authors:** Jonas Saal, Tobias Bald, Markus Eckstein, Manuel Ritter, Peter Brossart, Jörg Ellinger, Michael Hölzel, Niklas Klümper

**Affiliations:** Medical Clinic III for Oncology, Hematology, Immune-Oncology and Rheumatology, University Hospital Bonn, Bonn, Germany; Institute of Experimental Oncology, University Hospital Bonn, Bonn, Germany; Center for Integrated Oncology, Aachen/Bonn/Collogne/Düsseldorf (CIO-ABCD), Bonn, Germany; Institute of Experimental Oncology, University Hospital Bonn, Bonn, Germany; Center for Integrated Oncology, Aachen/Bonn/Collogne/Düsseldorf (CIO-ABCD), Bonn, Germany; Institute of Pathology, University Hospital Erlangen, Friedrich-Alexander-Universität Erlangen-Nürnberg (FAU), Erlangen, Germany; Comprehensive Cancer Center EMN, University Hospital Erlangen, Friedrich-Alexander-Universität Erlangen-Nürnberg, Erlangen, Germany; Center for Integrated Oncology, Aachen/Bonn/Collogne/Düsseldorf (CIO-ABCD), Bonn, Germany; Department of Urology and Pediatric Urology, University Hospital Bonn, Bonn, Germany; Medical Clinic III for Oncology, Hematology, Immune-Oncology and Rheumatology, University Hospital Bonn, Bonn, Germany; Center for Integrated Oncology, Aachen/Bonn/Collogne/Düsseldorf (CIO-ABCD), Bonn, Germany; Center for Integrated Oncology, Aachen/Bonn/Collogne/Düsseldorf (CIO-ABCD), Bonn, Germany; Department of Urology and Pediatric Urology, University Hospital Bonn, Bonn, Germany; Institute of Experimental Oncology, University Hospital Bonn, Bonn, Germany; Center for Integrated Oncology, Aachen/Bonn/Collogne/Düsseldorf (CIO-ABCD), Bonn, Germany; Institute of Experimental Oncology, University Hospital Bonn, Bonn, Germany; Center for Integrated Oncology, Aachen/Bonn/Collogne/Düsseldorf (CIO-ABCD), Bonn, Germany; Department of Urology and Pediatric Urology, University Hospital Bonn, Bonn, Germany

## Abstract

Static biomarkers like programmed death-ligand 1 (PD-L1) are insufficient to accurately predict response to immune checkpoint inhibition. Therefore, on-treatment biomarkers, which measure immediate therapy-associated changes, are currently shifting into the focus of immuno-oncology. A prime example of a simple predictive on-treatment biomarker is the early C-reactive protein (CRP) kinetics with its predictive CRP flare-response phenomenon. Here, we were able to confirm the predictive value of CRP flare-response kinetics in the pivotal phase III OAK trial (NCT02008227), which compared atezolizumab with docetaxel in patients with non-small cell lung cancer. Of note, CRP flare-response predicted favorable outcomes only in the immune checkpoint inhibition–treated subgroup, which suggests that it is an immunotherapy-specific phenomenon. In conclusion, we have for the first time validated the high predictive value of early CRP kinetics in a pivotal phase III trial, justifying the broad use of this cost-effective and easy-to-implement on-treatment biomarker to optimize therapy monitoring for patients with non-small cell lung cancer.

Most of the currently used predictive biomarkers for immune checkpoint inhibition (ICI), such as the intratumoral programmed death-ligand 1 (PD-L1) expression, are determined prior to initiation of therapy. Such static biomarkers may not be sufficient to accurately predict response to ICI because of the complexity and dynamics of antitumor immune responses (1). Therefore, on-treatment biomarkers, which measure immediate therapy-associated changes, are currently shifting into the focus of immuno-oncology. From a clinical point of view, when response prediction fails prior to therapy, rapid distinction between treatment success and failure is of immense importance, both to avoid therapy-related toxicity and to allow early adjustment to more effective therapies.

A prime example of a simple, cost-effective, and easy-to-implement predictive on-treatment biomarker is the early C-reactive protein (CRP) kinetics. We and others have recently shown that the CRP flare-response phenomenon, which is defined by an early CRP increase after initiation of ICI followed by a decrease below baseline, predicts ICI response across entities in metastatic renal cell carcinoma, urothelial cancer, and non-small cell lung cancer (NSCLC) (2-5).

However, to date, CRP flare-response kinetics has not been prospectively validated in a phase III clinical trial, which is needed prior to broad application in clinical practice.

##  

Data from the pivotal phase III OAK trial (NCT02008227) have been made available through vivli.org (6). Roche, which provided the data, and an independent review panel, which includes ethics, approved our post hoc analysis (request ID #7797). Data analysis was performed using R Studio (v.1.4). Patients were divided into the 3 CRP kinetics groups based on their on-treatment CRP levels as defined by Fukuda et al. (2): CRP flare-response, doubling of baseline CRP within first month and drop at least once within 3 months; CRP responders, at least 30% decrease of baseline CRP without prior flare; and the remaining patients as CRP non-responders. CRP was assessed according to local standards before each 21-day cycle, thus, once within the first month after ICI start (median = 22 days, interquartile range [IQR] = 22-22 days). CRP was measured in the accredited clinical laboratories of the study centers.

Of the 1225 patients who participated in the study, CRP kinetics could be analyzed in 758. Missing CRP values was the reason for study exclusion in 228 cases. Because the CRP kinetics definition considers the longitudinal CRP concentration within the first 3 months after the start of therapy, an additional 239 patients who died or left the study within this 3-month observation interval were excluded to avoid misclassification.

To compare the likelihood of achieving a complete or partial response for patients with CRP response or CRP flare-response compared with CRP non-response as the best overall response (BOR), we evaluated odds ratios (ORs). The BOR was defined as the best response recorded from the start of treatment until disease progression or recurrence.

##  

We validated the prognostic value of early on-treatment CRP kinetics in the pivotal phase III OAK trial (n = 758), which compared atezolizumab (anti-PD-L1 antibody) with docetaxel in patients with NSCLC after failure of platinum-containing chemotherapy (Figure 1). Importantly, CRP flare-response was associated with favorable outcomes only in atezolizumab-treated patients where it also occurred more frequently than in the docetaxel subgroup (11.0% vs 5.6%; Pearson χ^2^*P* = .0147). Individual CRP kinetics of the atezolizumab-treated patients stratified for the CRP kinetics subgroups is depicted in Supplementary Figure 1 (available online). Of note, patients with NSCLC on atezolizumab with CRP response (OR = 5.3; χ^2^*P* < .0001) or CRP flare-response (OR = 4.4; χ^2^*P* = .0001; Figure 1, D) kinetics had an approximately fivefold increased likelihood of objective treatment response (complete or partial response) as BOR compared with CRP non-responders. Of note, baseline characteristics did not differ between CRP kinetics subgroups (Supplementary Table 1, available online).

**Figure 1.**
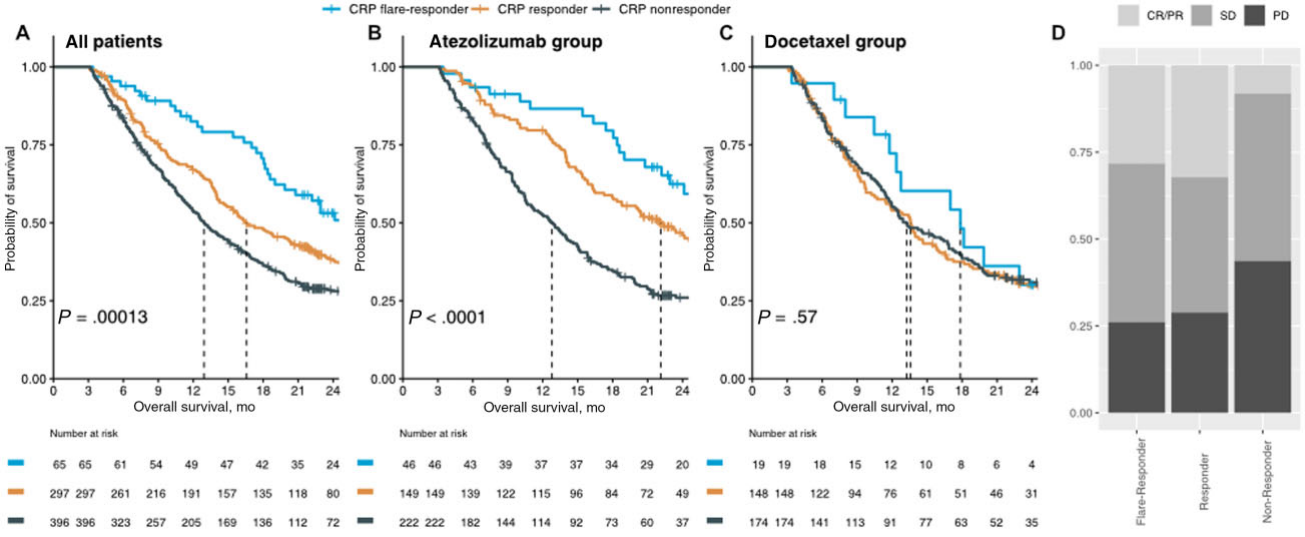
Early on-treatment CRP kinetics predicts outcome for patients with NSCLC within the phase III OAK trial. Kaplan-Meier survival curves showing overall survival (OS) after treatment initiation stratified according to CRP kinetics groups for the entire phase III OAK cohort **(A)**, as well as the atezolizumab- **(B)** and docetaxel-treated subgroup **(C)**. **D)** CRP flare-responders and responders are more likely to respond to atezolizumab as best overall response (BOR) compared with CRP non-responders. CR/PR = complete or partial response; CRP = C-reactive protein; NSCLC = non-small cell lung cancer; SD = stable disease; PD = progressive disease.

In univariable Cox regression, CRP flare-responders show a risk reduction of 48% (hazard ratio [HR] = 0.52, 95% confidence interval [CI] = 0.37 to 0.75; *P* < .001) for death compared with CRP non-responders. Besides CRP kinetics, histology (squamous vs non-squamous), PD-L1 status, and Eastern Oncology Cooperative Group (ECOG) performance status were associated with overall survival (Supplementary Table 2, available online). In multivariable Cox regression, CRP kinetics remained an independent predictor of survival after co-adjustment for histology, PD-L1 status, and ECOG (Supplementary Table 2, available online). In concordance, CRP kinetics provides independent predictive information to atezolizumab response in the PD-L1 negative and positive subgroups (Figure 2).

**Figure 2.**
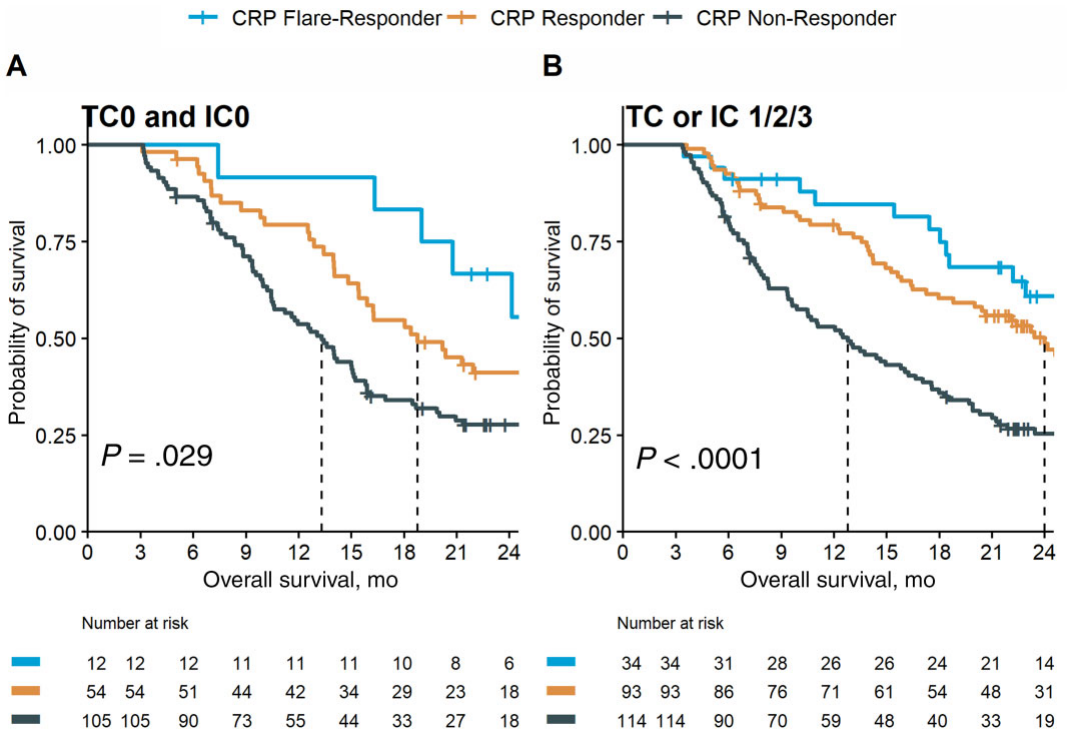
Early on-treatment CRP kinetics predicts outcome in atezolizumab-treated patients regardless of PD-L1 status. Kaplan-Meier survival curves showing overall survival (OS) after treatment initiation stratified according to CRP kinetics groups for the PD-L1–negative **(A)** and PD-L1–positive (≥1% PD-L1 on tumour cells [TC1/2/3] or tumour-infiltrating immune cells [IC1/2/3]) **(B)** atezolizumab-treated subgroup of the phase III OAK cohort. CRP = C-reactive protein; PD-L1 = programmed death-ligand 1.

Of note, 55.4% (36 of 65) of patients met the CRP flare-response criteria already after 6 weeks, and prognostic impact was already present 6 and 9 weeks after treatment start (Table 1). Thus, evaluation of early CRP kinetics may provide valuable predictive information before initial radiologic staging, which is usually performed at week 8-12.

**Table 1.** Univariable Cox regression results for early CRP kinetics assessed at different time points in relation to OS for the atezolizumab treated cohort^a^

Characteristic	No.	HR (95% CI)	*P*
CRP kinetics after 6 wk	406		
Non-responder		—	
Responder		0.69 (0.52 to 0.93)	.014
Flare-responder		0.44 (0.25 to 0.79)	.006
CRP kinetics after 9 wk	417		
Non-responder		—	
Responder		0.63 (0.48 to 0.82)	<.001
Flare-responder		0.38 (0.23 to 0.64)	<.001
CRP kinetics after 12 wk	417		
Non-responder		—	
Responder		0.56 (0.43 to 0.73)	<.001
Flare-responder		0.42 (0.27 to 0.65)	<.001

a CI = confidence interval; CRP = C-reactive protein; HR = hazard ratio; OS = overall survival.

##  

Early CRP kinetics provides valuable predictive information as early as 6 weeks after initiation of therapy and therefore opens a wide therapeutic window for early treatment adjustments [eg, in the setting of biomarker-stratified intervention studies (7)]. Within this randomized phase III clinical trial, we demonstrated for the first time that early CRP kinetics is an immunotherapy-specific phenomenon that had minor prognostic potential in the docetaxel-treated subgroup. This suggests that the CRP flare-response kinetics reflects the dynamic phase of an effective ICI-induced antitumor immune response. Whether CRP is the optimal serum marker to capture these antitumor immune kinetics needs to be investigated by future studies, ideally based on unbiased proteomic serum analyses (8). However, our study provides clear evidence for the potential of early on-treatment inflammatory biomarkers to predict immunotherapy response within the first weeks after therapy start.

In the phase III OAK study, only 11.0% of ICI-treated patients with NSCLC showed a CRP flare-response, whereas our previous data suggest a more frequent occurrence [eg, 26.9% of our immune monitoring of immune therapy (IMIT) NSCLC cohort (5)] of the CRP flare-response phenomenon. In the IMIT NSCLC cohort, in a substantial proportion of patients, the flare occurred already within the first 2 weeks of therapy, a time point where CRP was not measured within the OAK study. Therefore, several CRP flare-responders were likely missed in the OAK study, and we suggest that longitudinal CRP levels should be measured earlier in future studies.

Of note, we show that CRP kinetics can provide prognostic information in patients receiving immunotherapy independent of PDL-1 status and NSCLC histology and could thus be easily integrated in everyday clinical care. Performing a multivariable Cox regression to analyze the effects of multiple predictor variables on the time-to-event outcome variable is a commonly used statistical method in survival analysis. However, including predictors that are measured at baseline (eg, PD-L1) and longitudinally under therapy (as CRP kinetics) may introduce time-varying confounding, which can bias the estimates of the hazard ratios. This has to be considered for our multivariable Cox regression model depicted in Supplementary Table 2 (available online).

Because CRP is a serum parameter that can be measured in any certified laboratory at low cost, early CRP kinetics can also be used in low-income countries where imaging infrastructure as the main response prediction tool may be limited to identify patients at higher risk for disease progression.

In conclusion, we have for the first time validated the high predictive value of early CRP kinetics in a pivotal phase III trial, justifying the broad use of this cost-effective and easy-to-implement on-treatment biomarker to optimize therapy monitoring.

## Supplementary Material

pkad027_Supplementary_Data

## Data Availability

Data from the phase III OAK trial supporting our findings (NCT02008227) were provided by Hoffmann—La Roche via vivli by permission (Data Request ID 00007797) and are available upon request through Vivli, Inc (https://vivli.org/, vivli ID VIV00005794).
