# Supplementary material for: Early C-reactive protein kinetics predicts immunotherapy response in non-small cell lung cancer in the phase III OAK trial
Source: JNCI Cancer Spectr. 2023 Apr 2;7(2):pkad027. doi: 10.1093/jncics/pkad027 (PMC10121335; doi:10.1093/jncics/pkad027)
Supplement: pkad027_Supplementary_Data [file pkad027_supplementary_data.pdf]

**Supplementary Material**

**Supplementary Table 1**: Baseline Characteristics of the OAK cohort stratified by CRP kinetics

| Characteristic | Flare-Responder, N = 65[1] | Responder, N = 297[1] | Non-Responder, N = 396[1] | p-value[2] |
|---|---|---|---|---|
| Age Group 65 | | | | 0.5 |
| < 65 | 32 (49%) | 168 (57%) | 211 (53%) | |
| >= 65 | 33 (51%) | 129 (43%) | 185 (47%) | |
| sex | | | | 0.3 |
| female | 31 (48%) | 110 (37%) | 149 (38%) | |
| male | 34 (52%) | 187 (63%) | 247 (62%) | |
| ECOG Performance Score | | | | 0.083 |
| 0 | 34 (52%) | 115 (39%) | 177 (45%) | |
| 1 | 31 (48%) | 182 (61%) | 219 (55%) | |
| smoking history | | | | 0.7 |
| current | 10 (15%) | 48 (16%) | 61 (15%) | |
| never | 13 (20%) | 44 (15%) | 73 (18%) | |
| former | 42 (65%) | 205 (69%) | 262 (66%) | |
| PD-L1 expression | | | | 0.3 |
| TC0 and IC0 | 21 (32%) | 124 (42%) | 175 (44%) | |
| TC1/2/3 or IC1/2/3 | 44 (68%) | 169 (57%) | 218 (55%) | |
| UNKNOWN | 0 (0%) | 4 (1.3%) | 3 (0.8%) | |
| Pathology/Histology per eCRF | | | | 0.8 |
| NON-SQUAMOUS | 50 (77%) | 221 (74%) | 290 (73%) | |
| SQUAMOUS | 15 (23%) | 76 (26%) | 106 (27%) | |

[1] n (%)

[2] Pearson's Chi-squared test; Fisher's exact test

**Supplementary Table 2:** Univariable and multivariable Cox regression for CRP kinetics and baseline characteristics in relation to OS for the entire OAK cohort.

| Characteristic | N | HR[1] | 95% CI[1] | p-value |
|---|---|---|---|---|
| **univariate** | | | | |
| CRP_resp_12 | 758 | | | |
| Non-Responder | | — | — | |
| Responder | | 0.77 | 0.64, 0.92 | **0.005** |
| Flare-Responder | | 0.52 | 0.36, 0.74 | **<0.001** |
| PD-L1 score | 751 | | | |
| TC0 and IC0 | | — | — | |
| TC1/2/3 or IC1/2/3 | | 0.83 | 0.70, 0.99 | **0.043** |
| Sex | 758 | | | |
| F | | — | — | |
| M | | 1.17 | 0.98, 1.41 | 0.081 |
| Age Group 65 | 758 | | | |
| < 65 | | — | — | |
| >= 65 | | 1.01 | 0.85, 1.20 | >0.9 |
| Pathology/Histology per eCRF | 758 | | | |
| NON-SQUAMOUS | | — | — | |
| SQUAMOUS | | 1.67 | 1.38, 2.02 | **<0.001** |
| ECOG Performance Score | 758 | | | |
| 0 | | — | — | |
| 1 | | 1.50 | 1.25, 1.79 | **<0.001** |
| treatment | 758 | | | |
| docetaxel | | — | — | |
| atezolizumab | | 0.81 | 0.68, 0.96 | **0.017** |
| **multivariate** | | | | |
| CRP kinetics | 751 | | | |
| Non-Responder | | — | — | |
| Responder | | 0.75 | 0.62, 0.90 | **0.002** |
| Flare-Responder | | 0.55 | 0.38, 0.78 | **<0.001** |
| PD-L1 score | 751 | | | |
| TC0 and IC0 | | — | — | |
| TC1/2/3 or IC1/2/3 | | 0.84 | 0.70, 1.00 | 0.051 |
| Pathology/Histology per eCRF | 751 | | | |
| NON-SQUAMOUS | | — | — | |
| SQUAMOUS | | 1.62 | 1.33, 1.96 | **<0.001** |
| ECOG Performance Score | 751 | | | |
| 0 | | — | — | |
| 1 | | 1.48 | 1.24, 1.78 | **<0.001** |
| treatment | 751 | | | |
| docetaxel | | — | — | |
| atezolizumab | | 0.82 | 0.68, 0.98 | **0.026** |

[1] HR = Hazard Ratio, CI = Confidence Interval

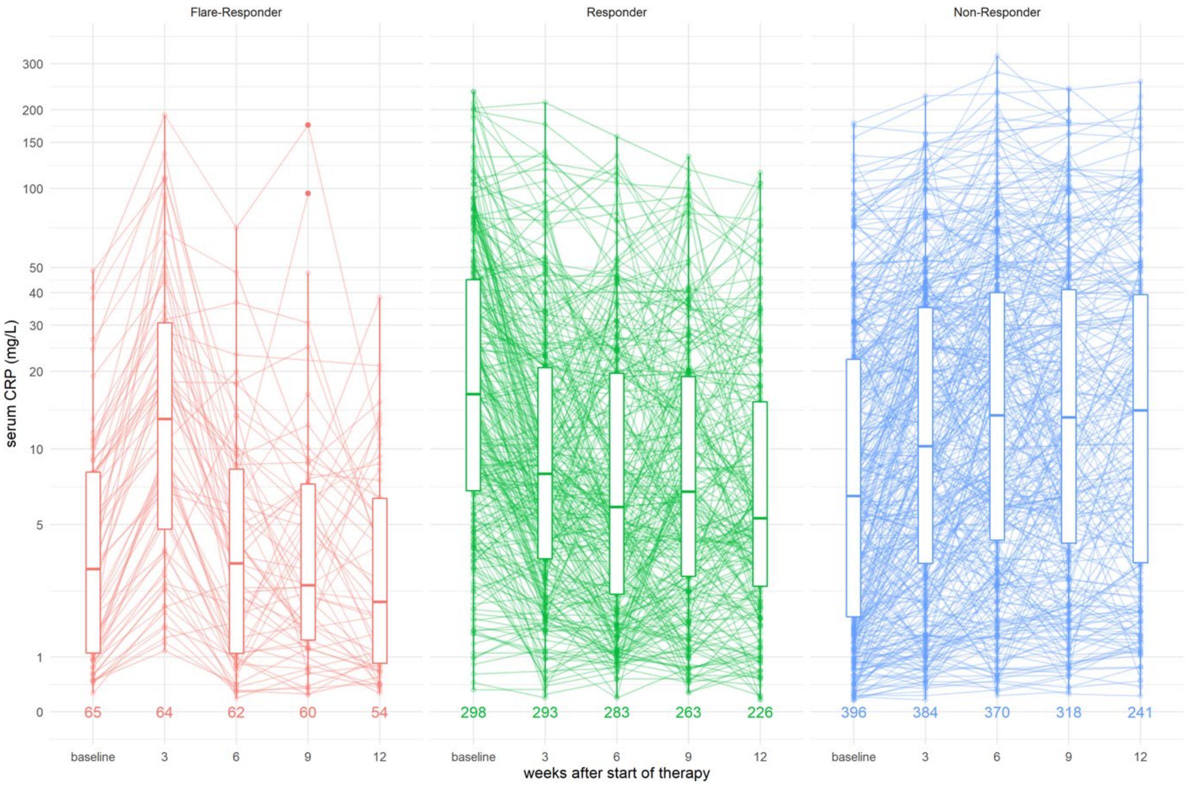


**Supplementary Figure 1: Individual CRP kinetics of the atezolizumab-treated patients stratified for the CRP-kinetics subgroups.** Individual CRP values are shown for the first three months, i.e., the observational period used for the CRP kinetics definition. Boxplots show median CRP and 1$^{st}$/3$^{rd}$ quartiles. Colored number at the bottom of the boxplots demonstrates number at risk.
